# Increasing incidence of type 1 diabetes in children and adolescents from 2012 to 2021 in Germany: trends before and during the COVID-19 pandemic

**DOI:** 10.1007/s10654-026-01417-y

**Published:** 2026-06-02

**Authors:** Carolin T. Lehner, Ivona Anastasova, Gunther Schauberger, Marian Eberl, Florian Schederecker, Martin Tauscher, Roman Gerlach, Stefanie J. Klug

**Affiliations:** 1 Chair of Epidemiology, TUM School of Medicine and Health, Technical University of Munich, Munich, Germany; 2 Institute for Medical Information Processing, Biometry and Epidemiology (IBE), LMU Munich, Munich, Germany; 3 Pettenkofer School of Public Health, Munich, Germany; 4 Bavarian Association of Statutory Health Insurance Physicians, Munich, Germany

**Keywords:** Type 1 diabetes, Children, Adolescents, Incidence trend, COVID-19 pandemic, Germany

## Abstract

Type 1 Diabetes (T1D) is a common chronic autoimmune disorder in children and adolescents worldwide. We described the development of T1D incidence from 2012 to 2021 and compared the incidence of the pre-pandemic period (2012–2019) with the pandemic period (2020–2021) in Bavaria, Germany. Routinely collected health claims from the Bavarian Association of Statutory Health Insurance Physicians (KVB), covering a population of 2 million children and adolescents (aged ≤ 19 years), were used. All cases of newly diagnosed T1D (ICD-10-GM E10) were included. Sex-specific annual and quarterly crude incidence rates (CIR) and age-standardized incidence rates (ASIR) were calculated. Sex-specific CIRs were calculated by 5-year age groups. Interrupted time series analysis was used to analyze trend changes in the pandemic versus the pre-pandemic period. From 2012 to 2021, 5,762 incident cases were identified in Bavaria. Overall, an increasing incidence was observed with an average annual increase of 3.7% in females (from 23.5 in 2012 to 32.5 per 100,000 person-years (py) in 2021) and 5.0% in males (from 25.2 to 38.9 per 100,000 py). The increase was highest in age groups 0–4 (12.4%) in males and 15–19 (8.5%) in females. Regression analysis showed no clear level or slope change in T1D incidence for both sexes during the pandemic period. When accounting for non-linear trends, a statistically significant level change was observed for females during the pandemic period (ß=7.66, 95% CI 0.23, 15.09), suggesting a potential association for females. The increasing T1D incidence in Bavarian youth from 2012 to 2021 highlights the importance of incidence surveillance.

**Supplementary Information:**

The online version contains supplementary material available at https://doi.org/10.1007/s10654-026-01417-y.

## Scientific background

Type 1 Diabetes (T1D) is a chronic health condition, characterized by autoimmune-mediated depletion of the insulin-producing beta cells of the pancreas, requiring comprehensive disease management and lifelong insulin therapy [1, 2]. Worldwide, 1.5 million children and adolescents (under 20 years of age) were affected by T1D in 2021, with incidences varying considerably by region and country [3]. In 2021, the second year of the pandemic, the highest absolute number of incident and prevalent T1D cases in children and adolescents (0–19 years) was found in Europe compared to other International Diabetes Federation regions [2]. Incidence rates were highest in Finland (52.2 cases per 100,000 population per year), Sweden (44.1 per 100,000 population per year), and Norway (33.6 cases per 100,000 population per year) [2]. Germany closely aligns with an estimated incidence rate of 33.6 per 100,000 persons reported for the same year [4].

Many European countries, including Germany, experienced an annual increase in T1D incidence of 3–7% among children (age 0–14) between 1989 and 2013 [5]. In Germany, T1D incidence has risen since the 1960s [6], and predictions indicate that prevalence is expected to double from 0.13% to 0.27% between 2006 and 2026 [7], highlighting the importance of monitoring. The German Diabetes Prospective Follow-up Registry (DPV) and registries from four of sixteen federal states report on national and regional trends on T1D-associated outcomes, respectively [8–10]. However, in Bavaria, the largest and second most populous federal state in Germany, studies on longitudinal T1D incidence are missing due to the lack of a federal registry. This constitutes an important gap as continuous monitoring and evaluation of T1D incidence trends are imperative for scientific understanding and healthcare management [11].

Assessing T1D incidence trends requires considering differences by sex and age [4], as well as time-varying factors such as the introduction of T1D screening and the COVID-19 pandemic. Since 2015, the Fr1da program has screened children for autoantibodies to detect pre-symptomatic T1D in Bavaria [12]. The impact of the COVID-19 pandemic on T1D incidence still remains unclear since international studies reported conflicting findings [13–15]. German studies comparing pre-pandemic and pandemic incidence are limited by focusing on the direct effect of the COVID-19 infection itself [16], missing statistical inference on the overall pandemic effect [4], the use of prescription-based data as a substitute for T1D diagnosis [17], as well as registry-based data that depend on voluntary reporting [18–20].

Therefore, the primary aim of this study is to analyze T1D incidence trends in Bavaria’s entire statutorily health-insured pediatric and adolescent population between 2012 and 2021. The secondary aim is to assess the effect of the pandemic on the development of T1D incidence by comparing the T1D incidence between the pre-pandemic period (2012–2019) and the pandemic period (2020–2021) using regression analysis.

## Methods

### Description of the study design, data source, and study population

This study constituted a secondary data analysis using routinely collected and anonymized outpatient health claims data provided by the Bavarian Association of Statutory Health Insurance Physicians (KVB). Therefore, the health claims data comprise all reported outpatient encounters, including routine medical visits, medical procedures, and medical events that are eligible for reimbursement and generate a diagnosis code. The KVB dataset encompasses the entire statutorily insured population in Bavaria, which includes 11 million people (around 85% of the Bavarian population). The study population consisted of children and adolescents aged 0–19 years with an incident diagnosis of T1D and residing in Bavaria during the study period between 2012 and 2021. The timeframe from 2012 to 2019 served as the pre-pandemic reference period, while 2020 to 2021 represented the pandemic period. Prior to data delivery, the KVB checked the data for plausibility based on the criteria of the claims schedule. Information on patients’ names, birth dates, and life-long insurance numbers was used to assign stable pseudonyms. Pseudonyms were excluded by the KVB if they consisted of changing values for name or birth date over time. For the purpose of analysis, anonymized health claims data were provided by the KVB, including aggregated information on sex (males, females), age group (0–4, 5–9, 10–14, 15–17, 18–19), calendar quarter (3-month calendar intervals), and 96 regional districts of Bavaria. Data on the population-at-risk were obtained from the German Federal Ministry of Health, providing official sex- and age-specific statistics of the statutorily insured population in Bavaria [21]. Prevalent T1D cases were excluded. Estimates on the population-at-risk per regional district were derived by the KVB based on patients’ residence districts in the claims data. This study was conducted according to the RECORD guideline for secondary data analyses [22] and in accordance with the “Good Practice of Secondary Data Analysis” guidelines [23].

### Description of eligibility criteria

This analysis focuses on children and adolescents to avoid misclassifying type 2 diabetes on insulin treatment as type 1 diabetes. Counting an incident case of T1D required the following eligibility criteria: (1) Only confirmed diagnoses according to the *International Classification of Diseases*,* Tenth Revision*,* German Modification* (ICD-10-GM) of T1D (Code E10) were included [24]. (2) Cases were included if they received the ICD-10-GM code E10 in at least two calendar quarters within four consecutive calendar quarters. The incident case was assigned to the calendar quarter in which the diagnosis appeared first. (3) Prevalent T1D cases were excluded, defined as cases diagnosed before the observation period. To avoid misclassifying prevalent cases as incident (e.g., in cases of new KVB database entries due to migration to Bavaria, relocation from other German states to Bavaria, or switch from private insurance to statutory insurance), cases were classified as prevalent if the calendar quarter in which a medical code was triggered for the first time and therefore leading to an first appearance in the KVB database occurs in the same calendar quarter as the first T1D code. (4) Cases with an additional diagnosis of type 2 diabetes (T2D) (ICD-10-GM Code E11) were excluded to avoid misclassifying type 2 diabetes as T1D.

### Statistical analysis

Descriptive statistics included calculating the size of the population-at-risk, the absolute number of new cases, the crude incidence rates (CIR) per 100,000 person-years (py), and the median age at diagnosis by sex and year. Age-standardized incidence rates per 100,000 py (ASIR) were calculated by direct age-standardization using the European Standard Population (2013 edition) [25]. Time series plots of CIR and ASIR were generated to describe T1D trends by sex (female, male) and age groups (0–4, 5–9, 10–14, 15–19) over time. The average annual change in incidence was calculated with the geometric mean of the annual growth rates.

### Interrupted time series

Through interrupted time series regression analysis (ITS), the time trend in quarterly ASIR of T1D per 100,000 person-years, stratified by sex, and its potential modifications by the COVID-19 pandemic were analyzed. Two regression models were used to fit ASIR in the pre-pandemic and pandemic segments. The main model 1 included the linear time trend, season (calendar quarters Q1 to Q3 compared to Q4), and the pandemic period. The pandemic effect encompassed both a level and a slope change in the pandemic period, as reports have proposed potential immediate and sustained associations of the pandemic period with T1D incidence [14]. A level change describes the immediate change in ASIR following the pandemic onset. A slope change signifies trend changes in the pandemic period compared to the pre-pandemic trend [26]. A separate model 2 was fitted based only on the pre-pandemic data (2012–2019), containing a linear trend and seasonal effects. Model 2 is used to predict counterfactual ASIR for the pandemic period (2020–2021) as if the pandemic never happened [26]. R² was used as the measure of goodness of fit. Assumptions of ordinary least squares regression were checked using graphical methods [27].

### Sensitivity analysis

For a sensitivity analysis of the ITS regression, in contrast to main model 1, a breakpoint in the linear time trend was identified using segmented linear regression, resulting in model 3 [28]. This approach aimed to account for the initial increasing ASIR and the subsequent decline during the pre-pandemic period (2012–2019). Model 4 constituted the corresponding counterfactual scenario. Analyses and graphical illustrations were performed in R 4.3.0 [29] using the packages included in the tidyverse framework [30]. Further, to investigate a potential age-specific pandemic effect on CIR, main model 1 was applied to the age groups 0–4, 5–9, 10–14, and 10–15 years for males and females.

## Results

### Descriptive results

The study population characteristics, including ASIR, CIR, and cases per year for both sexes, are presented in Table 1. In Bavaria, from 2012 to 2021, the population-at-risk covered approximately 2 million children and adolescents aged 0 to 19 years. In total, 5,762 new cases of T1D were identified (females: 2,584, 44.8%). The absolute number of cases and CIR increased over time. Compared to females, the male population-at-risk was consistently larger, and males consistently made up more than 50% of cases. Overall, the median age at diagnosis decreased for both sexes, by 0.49 years for females and 1.61 years for males, from 2012 to 2021.

**Table 1:** Characteristics of study population of children and adolescents aged 0–19 years, and incidence rates by sex and year from 2012 to 2021

	Population at risk (*n*)		New cases (*n*)		Median age (years)		CIR		ASIR	
Year	Female	Male	Female	Male	Female	Male	Female	Male	Female	Male
2012	947,124	998,596	217	249	11.26	12.12	22.9	24.9	23.5	25.2
2013	937,199	987,795	225	245	11.28	12.10	24.0	24.8	24.6	25.1
2014	936,018	986,728	244	288	11.24	11.30	26.1	29.2	27.0	30.0
2015	939,224	990,289	255	339	11.35	11.31	27.2	34.2	27.7	35.3
2016	945,026	998,049	272	323	10.69	10.77	28.8	32.4	30.1	33.2
2017	953,842	1,009,814	241	330	9.53	11.91	25.3	32.7	26.1	33.8
2018	956,622	1,012,710	249	313	10.82	11.62	26.0	30.9	26.8	31.9
2019	963,012	1,017,748	253	327	11.70	11.58	26.3	32.1	26.7	33.1
2020	971,073	1,025,290	315	361	9.87	11.02	32.4	35.2	33.2	36.2
2021	975,369	1,028,988	313	403	10.77	10.51	32.1	39.2	32.5	38.9

New case: incident cases of type 1 diabetes (T1D) per year, CIR: crude incidence rate per 100,000 person-years (py), ASIR: age-standardized incidence rate per 100,000 py (European Standard Population 2013), Median age: median age at diagnosis (years), n: numbers

### Incidence trends of type 1 diabetes

**Fig. 1:**
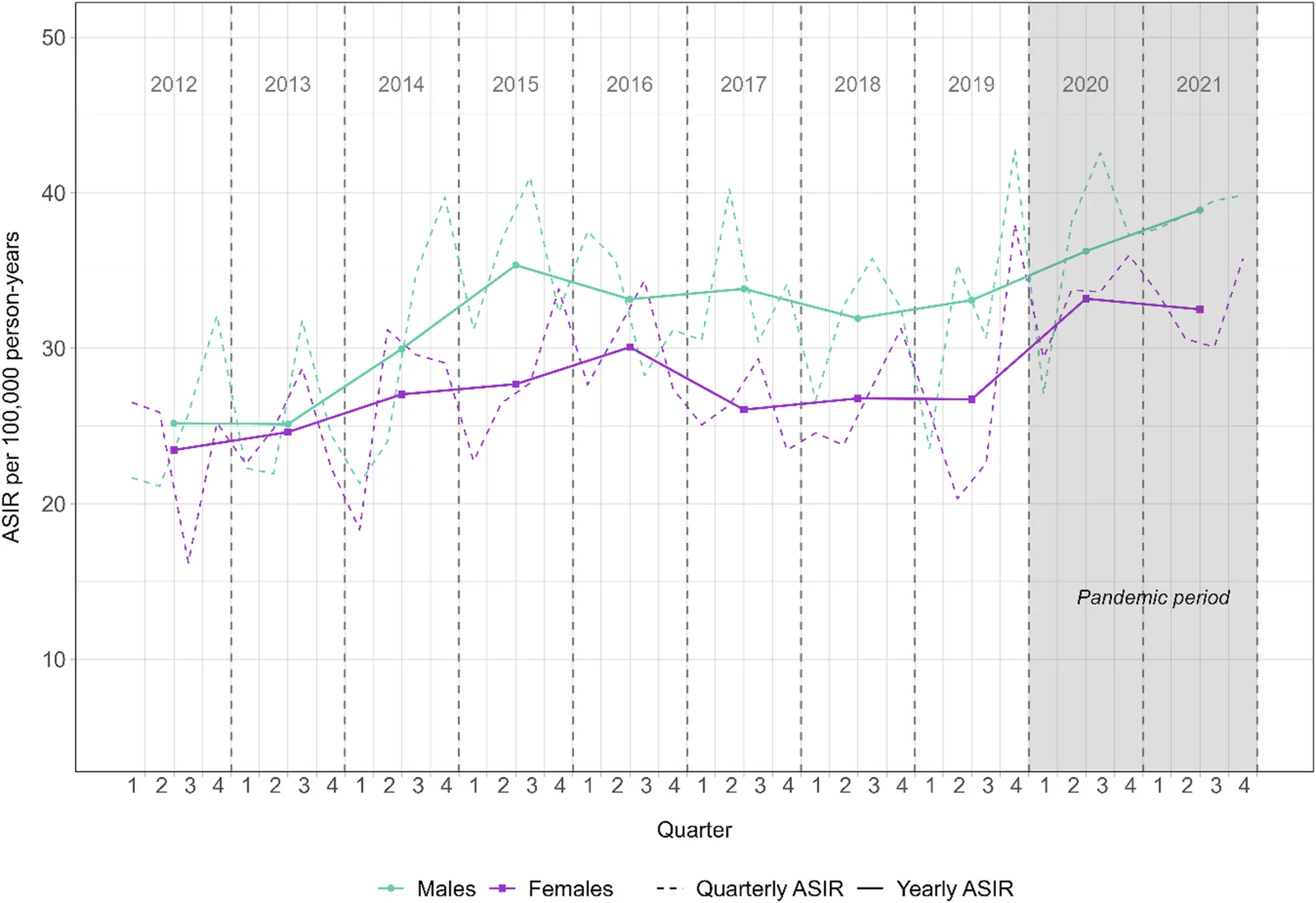
Time series analysis of age-standardized incidence rates (ASIR) per 100,000 person-years of type 1 diabetes (T1D) by sex, calendar quarter, and year. Grey shaded area: pandemic period, ASIR: age-standardized incidence rate per 100,000 person-years (European Standard Population 2013)

**Fig. 2:**
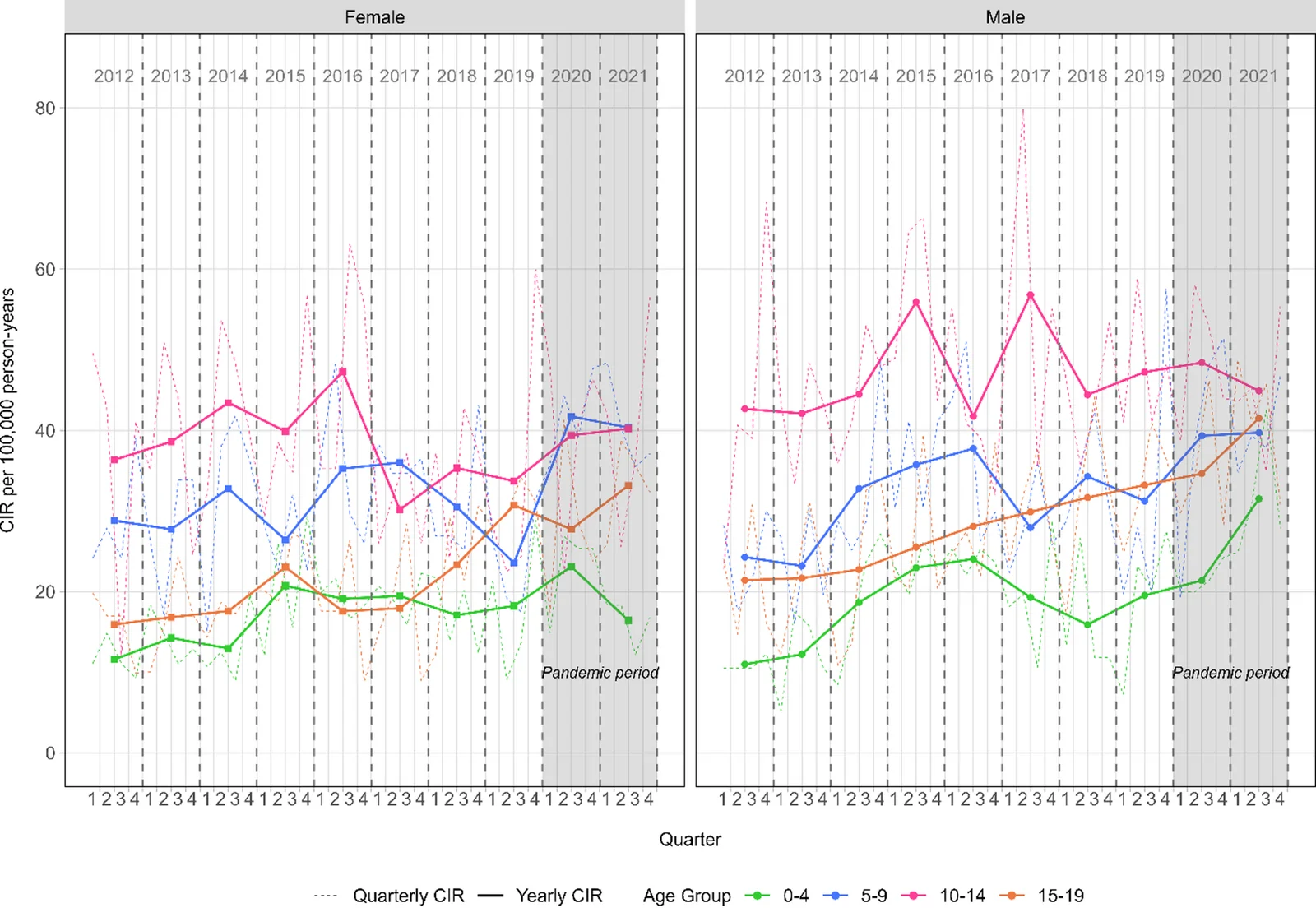
Time series analysis of crude incidence rates (CIR) per 100,000 person-years of type 1 diabetes (T1D) by sex, age group, calendar quarter, and year. Grey shaded area: pandemic period

The overall ASIR was 27.8 and 32.3 per 100,000 py in females and males, respectively, for the whole study period, and 26.6 and 30.9 per 100,000 py, respectively, for the pre-pandemic period (2012–2019). ASIR increased over time from 2012 to 2021, with consistently higher rates in males than females (Fig. 1). The increasing trend of ASIR during the pre-pandemic period was observed until 2015 for males and 2016 for females, followed by a decline until 2018 and 2019, respectively. Thereafter, ASIR increased again, peaking in 2021 for males and in 2020 for females during the pandemic period. ASIR increased by an annual average of 4.0% in males and 1.9% in females for the pre-pandemic period and by an annual average of 5.0% in males and 3.7% in females for the whole study period. From 2019 to 2020, ASIR increased 24.2% in females and 9.5% in males. This increase continued from 2020 to 2021 in males (7.3%) but not in females (−2.1%). Quarterly ASIR showed that overall peak ASIRs were reached before the start of the pandemic in Q4 2019 for both sexes (females: 38.0 per 100,000 py, males: 42.8 per 100,000 py) and Q3 2020 for males (42.6 per 100,000 py), while rates dropped in Q1 2020 in both sexes (Fig. 1, Supplementary Table S1). Age-specific rates are slightly higher for males than females and show a clear increase over time for all age groups, except for the 10–14 years age group for both sexes (Fig. 2). Overall, CIR increased with age, with the highest rates in age-group 10–14 years for both sexes (females: 38.5 per 100,000 py, males: 46.8 per 100,0000 py) and subsequently decreased in age group 15–19 for the whole study period (females: 22.2 per 100,000 py, males: 28.8 per 100,000 py, Supplementary Table S2). The average increase from 2012 to 2021 was lowest in the age group 10–14 years for both sexes (females: 1.1%, males: 0.6%) and highest in the age group 15–19 (8.5%) for females and in the age group 0–4 (12.4%) for males (Supplementary Table S2).

### Associations of the pandemic period and incidence of T1D

**Fig. 3:**
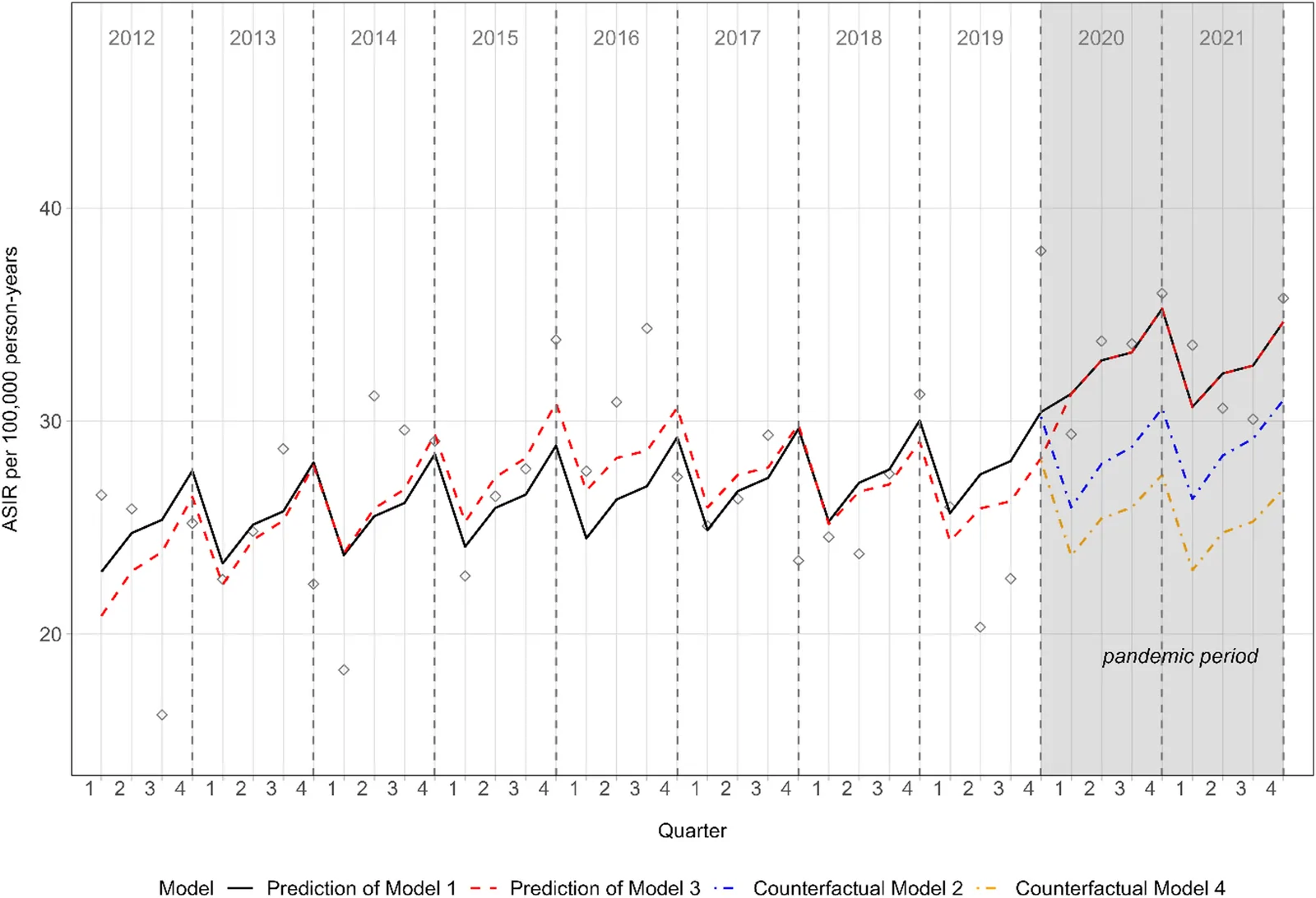
Interrupted time series regression analysis (2012–2021) of age-standardized incidence rates (ASIR) per 100,000 person-years of type 1 diabetes (T1D), females of ages 0–19. Rectangle points: observed ASIR. Black solid line: Model 1 adjusted for time trend, pandemic and seasonal effect. Blue dotted line: Counterfactual model 2 based on data from 2012–2019. Red dotted line: Model 3 (sensitivity analysis) adjusted for time trend, pandemic and seasonal effect and breakpoint in Q1 2016. Orange dotted line: Counterfactual model 4 (sensitivity analysis) based on data from 2012–2019 and breakpoint in Q1 2016. Grey shaded area: pandemic period

**Fig. 4:**
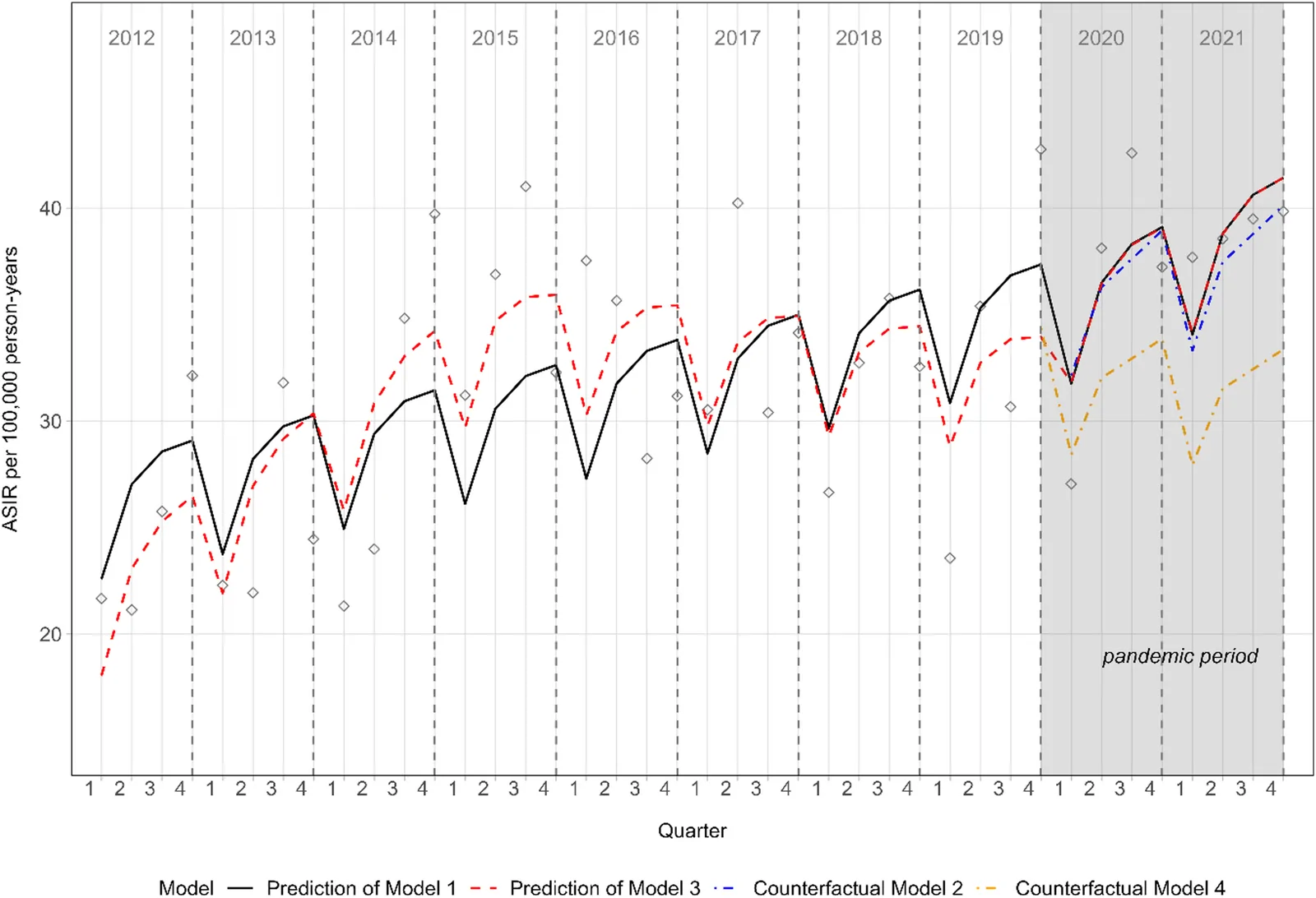
Interrupted time series regression analysis (2012–2021) of age-standardized incidence rates (ASIR) per 100,000 person-years of type 1 diabetes (T1D), males of ages 0–19. Rectangle points: observed ASIR. Black solid line: Model 1 adjusted for time trend, pandemic and seasonal effect. Blue dotted line: Counterfactual model 2 based on data from 2012–2019. Red dotted line: Model 3 (sensitivity analysis) adjusted for time trend, pandemic and seasonal effect and breakpoint in Q2 2015. Orange dotted line: Counterfactual model 4 (sensitivity analysis) based on data from 2012–2019 and breakpoint in Q2 2015. Grey shaded area: pandemic period

For females, the interrupted time series regression model 1 showed an increasing time trend in the pre-pandemic period (2012–2019) that was not statistically significant (ß=0.10, 95% CI −0.06, 0.25) (Fig. 3, Supplementary Table 3). For males, mean ASIR increased by 0.3 per 100,000 py per calendar quarter (95% CI 0.10,0.49) during the pre-pandemic period (Fig. 4, Supplementary Table 3). Additionally, during the pandemic period (2020–2021), no statistically significant change in slope in females (ß=−0.25, 95% CI −1.58, 1.07) and males (ß=0.28, 95% CI −1.35, 1.91) was observed. Similarly, there was no statistically significant level change in females (ß=5.46, 95% CI −1.82, 12.75) and males (ß=−0.56, 95% CI −9.51, 8.39) (Supplementary Table S3). Seasonal effects revealed lower ASIR in Q1, Q2, and Q3 compared to Q4, though this was only statistically significant for Q1 (females: ß=−4.45, 95% CI −8.28, −0.61; males: ß =−5.62, 95% CI −10.33, −0.91). Model 1 accounted for 41% and 49% of the variance in females and males, respectively. Diagnostic plots revealed no relevant violations of the Ordinary Least Squares assumption (Supplementary Figures S1 and S2). Figures 3 (females) and 4 (males) illustrate predicted ASIR in 2020–2021, taking into account potential pandemic effects (model 1) and counterfactual ASIR that continue the pre-pandemic trend (model 2) to predict ASIR in the absence of the pandemic.

### Sensitivity analysis

In the sensitivity analysis of the segmented regression analysis of the temporal trend in T1D ASIR, a breakpoint was added to main model 1 to capture nonlinear trends during the pre-pandemic period. The breakpoint was detected in Q1 2016 for females and in Q2 2015 for males (model 3). In both sexes, a statistically significant positive increase in ASIR until Q2 2015 for males and Q1 2016 for females was identified (females: ß=0.37, 95% CI 0.04, 0.70; males: ß=0.97, 95% CI 0.49, 1.45), followed by a negative slope change until Q4 2019 (females: ß=−0.57, 95% CI −1.18, 0.05; males: ß=−1.09, 95% CI −1.82, −0.36), leading to a decreasing incidence trend in this segment. Afterwards, a positive, not statistically significant, pandemic slope change between Q1 2020 and Q4 2021 was identified (females: ß=0.04, 95% CI −1.28,1.36; males: ß=0.71, 95% CI −0.78, 2.19). By summation of these coefficients, ASIR decreased by 0.16 per 100,000 py per calendar quarter in females and increased by 0.59 per 100,000 py per calendar quarter in males in the pandemic segment. The level change in model 3 was statistically significant in females (ß=7.66, 95% CI 0.23, 15.09), indicating an immediate upward shift in ASIR in Q1 2020. In males, the level change was not statistically significant (ß=2.80, 95% CI −5.51, 11.11). Compared to main model 1, the R² of model 3 increased to 47% in females and 60% in males, however this increase might reflect the inclusion of the additional breaking point. Summaries of models 1 and 3 are included in the supplementary materials (Supplementary Tables S3 and S4). Age-specific regression analysis revealed no statistically significant slope or level change of CIR during the pandemic for males (Supplementary Table S5). For females, a statistically significant level change for the age group 5–9 years with a wide confidence interval was observed (ß= 14.25, 95% CI 0.29, 28.21), suggesting an immediate increase in CIR in Q1 2020 (Supplementary Table S5).

## Discussion

This analysis of nearly 2 million statutory health-insured children and adolescents aged up to 19 years residing in Bavaria, Germany, showed an increasing T1D incidence from 2012 to 2021 in both sexes. In the pre-pandemic period (2012–2019), ASIR increased by an annual average of 1.9% in females and 4.0% in males. When including the pandemic period (2012–2021), the annual average increase of ASIR almost doubled in females, reaching 3.7% and increased by 25% in males, reaching 5.0%. This clear increasing trend was also observed for all age groups except for the age group 10–14 years. Average annual increases were highest in the age group 15–19 in females (8.5%) and 0–4 in males (12.4%), and lowest in the 10–14 age group for both sexes (1.1% in females and 0.6% in males). Interrupted time series regression showed an increasing trend from 2012 to 2019 for both sexes. No slope and level change in T1D incidence could be identified during the first two years of the pandemic period for both sexes. When accounting for non-linear trends, a statistically significant level change in ASIR for females in Q1 2020 suggested a possible association with the pandemic onset, although the magnitude of the association remains uncertain due to the wide confidence intervals. Age-specific regression analysis showed a statistically significant level change in CIR for females in the age group 5–9 years; however, the strength of the association with the pandemic onset remains unclear due to the wide confidence interval.

### Trends in T1D incidence

The observed increasing T1D incidence trend in children and adolescents in our study aligns with the upward trend documented in Europe [5] and worldwide [31]. A study across 22 European countries, using a mixed effects Poisson regression model, reported an average annual increase between 1989 and 2013 of 3.4% with sex-adjusted annual increases of 2.6% to 4.4% in German study centers [5]. Similarly, our study found annual increases in ASIR of 1.9% in females and 4.0% in males (2012–2019). Our study results align with findings of higher incidence in males than females worldwide (age 15–39 years) [32], as well as increasing overall incidence up to age 14, which declines in the age group 15–19 [32]. In our study, the annual increase was highest in age groups 0–4 for males and 15–19 for females. A similar trend in the youngest age group, 0–4 years, has been observed in other studies [5, 33]. A potential reason here may be earlier disease onset, rather than increased overall risk [34, 35], supported by findings of a reduced median time from autoantibody appearance to T1D onset [36]. In our analysis, age at diagnosis decreased by 0.49 years in females and by 1.61 years in males from 2012 to 2021, suggesting earlier disease onset or screening effects. The implications are important, as an earlier disease onset could increase children’s risk for comorbidities [37], raise mortality [37], and increase the burden on healthcare systems [38, 39]. Studies from Germany consistently report increasing T1D incidence trends [4, 18–20]. An analysis of approximately 1 million children and adolescents (< 18 years) from a research database containing routine health care data from selected health insurance providers across Germany, including Bavaria, supports our result as it also shows an increase in incidence from 2015 to 2021 with slightly lower rates of 24.9 to 30.8 per 100,000 persons in females and 26.5 to 36.2 per 100,000 persons in males compared to our analysis [4]. Our analysis added three more years of observation, mitigated potential selection bias by covering the entire Bavarian statutory insured population, and modelled pandemic effects. Additionally, data from the diabetes surveillance in Germany, conducted by the Robert Koch Institute, reported similar but slightly lower incidence rates between 2014 and 2022 to our analysis [40]. Kamrath et al. using nation-wide incidence, from the DPV Registry covering over 90% of the pediatric population diagnosed with type 1 diabetes within Germany, including Bavaria, also showed an increase from approximately 16 to 23 per 100,000 py between 2011 and 2021, with lower annual increases of 2.7% in males and a similar increase of 2.0% in females (2011–2019) compared to our analysis [19]. A study from the RKI, also using nationwide data from the DPV Registry, reported coverage-corrected national incidence, showed no clear T1D incidence trend from 2014 to 2020, but a significant increase only in males in 2020 [18]. However, since German diabetes registries rely on voluntary reporting of the participating centers, time-varying changes in coverage rate and data quality are possible [18]. Despite the fact that regional differences in diabetic care for children and adolescents exist [41], these studies did not report regional trends in ASIR by federal state [4, 19, 20]. Our analysis found slightly higher incidence rates and average annual increases in Bavaria, particularly in boys, compared to national estimates, while our data do not include privately insured children and adolescents, calling for improved reporting of regional outcomes. This may encourage considerations of a Bavarian population-based T1D registry to enhance epidemiological monitoring, provide insight into patient care [42], and prevent diabetes-related complications [43]. Reasons for the observed increasing T1D incidence trend in children and adolescents remain unclear, but factors such as nutrition during infancy [44], obesity [45, 46], and maternal factors [47] have been explored as potential drivers of T1D incidence. However, causal links have not yet been established. Potentially, preventive measures such as increased screening efforts may lead to an increase in reported incidence.

### Association of the COVID-19 pandemic and T1D incidence

Comparing the T1D incidence during the pandemic (2020–2021) to the pre-pandemic period (2012–2019), the regression model adjusted for pandemic effects found no immediate pandemic impact. However, after accounting for a non-linear effect at Q1 2016 for females and at Q2 2015 for males, a statistically significant level change at Q1 2020 was detected in females, although the wide confidence interval indicates some remaining uncertainty regarding the magnitude of the association. Additionally, a statistically significant level change in females for the age group 5–9 years was observed, although the strength of the association remains unclear due to the wide confidence interval. Further, our results should be interpreted cautiously, due to the limited pandemic coverage up to 2021. The impact of the pandemic on incidence varied across the regions, while reports from Europe noted a decline in new cases in early 2020 [48, 49], a report from Germany, including Bavaria data, found no impact in Germany from January to May 2020 [19]. During the first year of the pandemic from 2020 to 2021, no sustained changes in incidence trends were observed in our analysis, consistent with Polish findings [48] and a meta-analysis of 55 countries (2017–2022) [15]. Also, when accounting for non-linear trends in our analysis, no slope change was observed, indicating no sustained changes during the first two years of the pandemic. However, it is important to note that our analysis is limited to the first two years of the pandemic; therefore, the interpretation of these findings should be cautious. In contrast, other meta-analyses covering global incidence trends reported significant increases of T1D incidence during the first year of the pandemic [13] and a sustained 27% increase during the second year of the pandemic in youth (< 19 years) compared to the pre-pandemic period in Europe, North America, the Middle East, Asia, and Australia [14]. Some German studies suggest significant increases from June to September 2020 and from March to June 2021 [19]. Since many studies investigating the impact of the COVID-19 pandemic on T1D incidence did not include sex-specific analysis [13, 14, 17, 50], our analysis adds sex as a possible modifier of the pandemic effect. While the regression analysis in our study showed no slope or level change during the first two years of the pandemic, when accounting for non-linear trends, a potential level change in females was shown, suggesting a potential association in females, with a wide confidence interval indicating an uncertain magnitude of the association. In contrast, Kamrath et al. found no sex-specific differences in observed versus expected incidence rates in German youth (< 18 years) [19], while a Finnish study reported significant increases only in males, based on 785 T1D cases from the Finnish Pediatric Diabetes Register (< 14 years) [51]. Reasons for the increasing incidence during the pandemic period may not be directly caused by the COVID-19 infection itself [52, 53] but rather be linked to risk factors for T1D exacerbated by lockdown-induced changes in lifestyle. Since the clinical presentation of type 1 diabetes symptoms is usually preceded by a presymptomatic stage, which can be detected by the presence of islet autoantibodies, the time to manifestation of type 1 diabetes can take weeks to years [54]. In Germany, increased body weight [55–57], a potential accelerator of T1D pathogenesis [34, 58], was reported, as well as increased mental health diseases in children and adolescents during the COVID-19 pandemic [59, 60]. Another reason might be a decline in common respiratory infections compared to pre-pandemic years in Bavaria [61], which may have influenced trends in both directions - reducing viral-mediated cases or increasing incidence due to reduced viral exposure [62]. A recent systematic review and meta-analysis linked a SARS-CoV-2 infection in children and adolescents to higher T1D risk in the United States but not in Europe [63], potentially accelerating disease onset in pre-symptomatic youth [64]. However, a Bavarian study also using health claims data from the KVB derived a 57% increased hazard ratio for T1D in children diagnosed with COVID-19 in 2020/2021 [16].

### Strengths and limitations

Our analysis describes the 10-year trend in T1D incidence using routinely collected health claims data of nearly 2 million children and adolescents in Bavaria, enabling a more robust analysis compared to selective-insurance studies. Using interrupted time series regression in our study allowed for associations with the pandemic period. However, the results must be interpreted in view of potential limitations. Health claims data collected for reimbursement purposes may not fully reflect the disease burden. The observed lower T1D incidence rates in most years in Q1 compared to Q4 could potentially reflect billing patterns that lead to more diagnoses toward the end of the year. Also, the peak of the incidence rate in Q4 in 2019 might be driven by monetary incentives introduced in September 2019 by the German Appointment Service and Supply Act, encouraging doctors to accept more new patients [65]. Additionally, diagnoses were not validated through insulin prescriptions, although analyses on routine data have shown potential misclassification in up to 30% of cases initially diagnosed as T1D [66]. We addressed this by only including confirmed cases with the same ICD-10 codes of T1D in two calendar quarters within four subsequent calendar quarters. Furthermore, cases with a concurrent T2D diagnosis were excluded to prevent misclassification of T2D as T1D, which potentially has led to the exclusion of actual T1D cases [67]. Also, undiagnosed subclinical cases were not captured since diagnosis usually relies on individuals seeking medical attention following the onset of symptoms [68]. However, this has been ameliorated by population-based voluntary screening in Bavaria since 2015, identifying 0.3% of approximately 169,446 screened children with pre-symptomatic T1D until 2022 [12]. Additionally, privately insured children and adolescents were not accounted for in these data. However, the impact of the socioeconomic gradient and the underestimation of the incidence is expected to be small as approximately 10,5% of the German population is privately insured [69], of whom 16% are children and adolescents aged under 20 years (in 2016), accounting for approximately 9% of privately insured children in Germany [70]. With only eight pandemic calendar quarters included in the regression analysis and predominantly wide confidence intervals, results should be interpreted cautiously. Lastly, data were provided on an aggregated level, therefore limiting the possibility of causal inference.

## Conclusion

In Bavaria, we observed increasing T1D incidence rates in children and adolescents from 2012 to 2021 in males and females as well as across age groups, except for the age group 10–14 years. Although the average annual increase in incidence accelerated in the pandemic period, no slope change during the COVID-19 pandemic could be found. But when accounting for non-linear trends, a potential level change of the ASIR in the COVID-19 pandemic was observed for females, while the magnitude of the association remains uncertain due to a wide confidence interval. Studies covering a longer time frame after the onset of the COVID-19 pandemic are needed to assess both the direct and indirect effects of the COVID-19 pandemic and to investigate the sex-specific impact of the pandemic on T1D incidence. Our results reveal a high and further increasing T1D incidence in Bavaria, emphasizing the need for continued surveillance of epidemiological trends in T1D to understand its burden in children and adolescents. Moreover, efforts to investigate the underlying factors contributing to the increasing incidence rates in T1D and to develop effective preventive strategies remain critical to mitigating the burden of disease.

## Supplementary Information

Below is the link to the electronic supplementary material.

Supplementary Material 1
