# Supplementary material for: Increasing incidence of type 1 diabetes in children and adolescents from 2012 to 2021 in Germany: trends before and during the COVID-19 pandemic
Source: Eur J Epidemiol. 2026 Jun 2;41(7):881–92. doi: 10.1007/s10654-026-01417-y (PMC13572331; doi:10.1007/s10654-026-01417-y)
Supplement: Supplementary file 1 [file 10654_2026_1417_MOESM1_ESM.docx]

**Electronic supplementary material**

**Journal:** European Journal of Epidemiology

**Increasing incidence of type 1 diabetes in children and adolescents from 2012 to 2021 in Germany: Trends before and during the COVID-19 pandemic**

Carolin T. Lehner^1^, Ivona Anastasova^1,2,3^, Gunther Schauberger^1^, Marian Eberl^1^, Florian Schederecker^1^, Martin Tauscher^4^, Roman Gerlach ^4^, Stefanie J. Klug^1^

1 Technical University of Munich, TUM School of Medicine and Health, Chair of Epidemiology, Munich, Germany

2 Institute for Medical Information Processing, Biometry and Epidemiology (IBE), LMU Munich, Germany
3 Pettenkofer School of Public Health, Munich, Germany
4 Bavarian Association of Statutory Health Insurance Physicians, Munich, Germany

**Correspondence address:**

Carolin Lehner

Technical University of Munich, TUM School of Medicine and Health, Chair of Epidemiology

Am Olympiapark 11

80809 Munich, Germany

carolin.lehner@tum.de

**Supplementary Table S1** Age-standardized incidence rates (ASIR) of type 1 diabetes (T1D) per 100,000 person-years (py) by calendar quarter and sex (European Standard Population 2013)

| **Sex** | **Quarter** | **2012** | **2013** | **2014** | **2015** | **2016** | **2017** | **2018** | **2019** | **2020** | **2021** |
| --- | --- | --- | --- | --- | --- | --- | --- | --- | --- | --- | --- |
| female | Q1 | 26.5 | 22.6 | 18.3 | 22.7 | 27.7 | 25.1 | 24.6 | 26.0 | 29.4 | 33.6 |
| female | Q2 | 25.9 | 24.8 | 31.2 | 26.5 | 30.9 | 26.3 | 23.8 | 20.3 | 33.8 | 30.6 |
| female | Q3 | 16.2 | 28.7 | 29.6 | 27.8 | 34.4 | 29.3 | 27.5 | 22.6 | 33.6 | 30.1 |
| female | Q4 | 25.2 | 22.3 | 29.1 | 33.8 | 27.4 | 23.4 | 31.3 | 38.0 | 36.0 | 35.8 |
| male | Q1 | 21.7 | 22.3 | 21.3 | 31.2 | 37.5 | 30.5 | 26.7 | 23.6 | 27.1 | 37.7 |
| male | Q2 | 21.1 | 21.9 | 24.0 | 36.9 | 35.7 | 40.2 | 32.7 | 35.4 | 38.1 | 38.6 |
| male | Q3 | 25.8 | 31.8 | 34.8 | 41.0 | 28.2 | 30.4 | 35.8 | 30.7 | 42.6 | 39.5 |
| male | Q4 | 32.1 | 24.5 | 39.7 | 32.3 | 31.2 | 34.1 | 32.6 | 42.8 | 37.2 | 39.8 |

**Supplementary Table S2** Overall crude incidence rates (CIR) of type 1 diabetes (T1D) per 100,000 person-years (py) and average annual changes in % by age group, sex, and time period

|  |  | **CIR per 100,000 py** | | **Average annual change (%)** | |
| --- | --- | --- | --- | --- | --- |
| **Sex** | **Age group** | **2012-2019** | **2012-2021** | **2012-2019** | **2012-2021** |
| female | 0-4 | 16.8 | 17.5 | 6.7 | 4.0 |
| female | 5-9 | 30.1 | 32.5 | -2.8 | 3.8 |
| female | 10-14 | 38.1 | 38.5 | -1.1 | 1.1 |
| female | 15-19 | 20.3 | 22.2 | 9.8 | 8.5 |
| male | 0-4 | 18.1 | 19.9 | 8.6 | 12.4 |
| male | 5-9 | 30.9 | 32.8 | 3.7 | 5.6 |
| male | 10-14 | 46.8 | 46.8 | 1.5 | 0.6 |
| male | 15-19 | 26.7 | 28.8 | 6.5 | 7.6 |

**Supplementary Table S3** Time series regression model 1 of quarterly age-standardized incidence rates (ASIR) of type 1 diabetes (T1D) per 100,000 person-years (py) and factors of time by sex

|  | **Outcome: Quarterly ASIR of T1D (model 1)** | |
| --- | --- | --- |
|  | **Females**  **Coefficient (95% CI)** | **Males**  **Coefficient (95% CI)** |
| Constant | 27.27^***^ (23.31, 31.23) | 27.90^***^ (23.04, 32.77) |
| Pre-pandemic time trend | 0.10 (-0.06, 0.25) | 0.30^***^ (0.10, 0.49) |
| Quarter 1 | -4.45^**^ (-8.28, -0.61) | -5.62^**^ (-10.33, -0.91) |
| Quarter 2 | -2.73 (-6.50, 1.05) | -1.46 (-6.10, 3.18) |
| Quarter 3 | -2.20 (-5.94, 1.54) | -0.22 (-4.82, 4.38) |
| Level change | 5.46 (-1.82, 12.75) | -0.56 (-9.51, 8.39) |
| Slope change | -0.25 (-1.58, 1.07) | 0.28 (-1.35, 1.91) |
| Observations | 40 | 40 |
| R^2^ | 0.41 | 0.49 |
| Adjusted R^2^ | 0.30 | 0.40 |
| Residual Standard Error (df = 33) | 4.10 | 5.04 |
| F Statistic (df = 6; 33) | 3.85^***^ | 5.26^***^ |
|  | *p<0.1; **p<0.05; ***p<0.01 | |

**Supplementary Table S4** Time series regression model 3 of quarterly age-standardized incidence rates (ASIR) of type 1 diabetes (T1D) per 100,000 person-years (py) and factors of time by sex

|  | **Outcome: Quarterly ASIR of T1D (model 3)** | |
| --- | --- | --- |
|  | **Females**  **Coefficient (95% CI)** | **Males**  **Coefficient (95% CI)** |
| Constant | 24.97^***^ (20.39, 29.55) | 22.61^***^ (17.00, 28.21) |
| Trend | 0.37^**^ (0.04, 0.70) | 0.97^***^ (0.49, 1.45) |
| Slope change following Q2 2015 | - | -1.09^***^ (-1.82, -0.36) |
| Slope change following Q1 2016 | -0.57^*^ (-1.18, 0.05) | - |
| Quarter 1 | -4.48^**^ (-8.19, -0.78) | -5.53^**^ (-9.75, -1.31) |
| Quarter 2 | -2.75 (-6.40, 0.90) | -1.47 (-5.62, 2.68) |
| Quarter 3 | -2.21 (-5.83, 1.40) | -0.23 (-4.34, 3.89) |
| Level change | 7.66^**^ (0.23, 15.09) | 2.80 (-5.51, 11.11) |
| Slope change following pandemic onset | 0.04 (-1.28, 1.36) | 0.71 (-0.78, 2.19) |
| Observations | 40 | 40 |
| R^2^ | 0.47 | 0.60 |
| Adjusted R^2^ | 0.35 | 0.52 |
| Residual Standard Error (df = 32) | 3.96 | 4.50 |
| F Statistic (df = 7; 32) | 4.04^***^ | 6.98^***^ |
|  | *p<0.1; **p<0.05; ***p<0.01 | |

**Supplementary Table S5** Time series regression model 1 of quarterly crude incidence rates (CIR) of type 1 diabetes (T1D) per 100,000 person-years (py) and factors of time by sex and age-groups

|  | **Males** | | **Females** | |
| --- | --- | --- | --- | --- |
|  | **Coefficient**  **(95% CI)** | | **Coefficient**  **(95% CI)** | |
| **Age groups** | **Slope change** | **Level change** | **Slope change** | **Level change** |
| 0-4 years | 1.80*  ( -0.26, 3.86) | -4.56  (-15.88, 6.76) | -1.41*  (-3.05, 0.24) | 4.87  (-4.18, 13.92) |
| 5-9 years | 0.89  (-2.21,3.99) | -0.59  (-17.63, 16.46) | -0.71  (-3.25, 1.83) | 14.25**  (0.29, 28.21) |
| 10-14 years | -0.73  (-4.50, 3.04) | -0.87  (-21.59, 19.85) | 0.48  (-3.44, 4.40) | 3.56  (-17.97, 25.08) |
| 15-19 years | -0.17  (-2.22,1.88) | 2.41  (-8.86, 13.67) | 0.80  (-1.08, 2.68) | -1.39  (-11.74, 8.95) |
|  |  | *p<0.1; **p<0.05; ***p<0.01 | | |

**Supplementary Figure S1** Assumptions of ordinary least squares regression analysis of model 1– diagnostic plots, females

**Supplementary Figure S2** Assumptions of ordinary least squares regression analysis of model 1 – diagnostic plots, males
